# The Influence of an Unsupported Sleeper on the Vertical Bearing Characteristics of Heavy-Haul Railway Ballast

**DOI:** 10.3390/ma17061434

**Published:** 2024-03-21

**Authors:** Dan Liu, Chengguang Su, Dawei Zhang, Caihao Lan

**Affiliations:** 1 School of Highway, Chang’an University, Xi’an 710064, China; liudan88r@chd.edu.cn; 2 China Railway First Survey and Design Institute Group Co., Ltd., Xi’an 710043, China; suchengguang.yy@crcc.cn; 3 School of Automobile, Chang’an University, Xi’an 710064, China; 4 MOE Key Laboratory of High-Speed Railway Engineering, Southwest Jiaotong University, Chengdu 610031, China; lancaihao5@163.com

**Keywords:** ballast, unsupported sleeper, discrete element method, vertical bearing characteristics, wheel/rail interaction

## Abstract

In order to study the influence of an unsupported sleeper on the vertical bearing characteristics of heavy-haul railway ballast, a three-dimensional discrete element model (DEM) was established for a ballasted track, by removing ballast particles that come into contact with the bottom of the sleeper from the model to simulate the unsupported sleeper. Vertical bearing characteristics for ballast on different types of unsupported sleepers were studied. The results showed that an unsupported sleeper could reduce the bearing area of the ballast below the sleeper and reduce the number of ballast particles that were in contact. It could also lead to an increase in the maximum contact force between the particles, accelerating the deterioration of the particles (thus affecting the overall performance of the ballast) and reducing the vertical stiffness of the ballast. As the unsupported length and width increased, vertical stiffness gradually decreased. The vertical ballast stiffness for an unsupported sleeper was then used in a dynamic coupled vehicle/track model, and the effect of the unsupported sleeper on wheel/rail interaction was analyzed. Results showed that increasing the unsupported length and width leads to a decrease in the supporting force on the unsupported sleeper and to an increase in the supporting force on the adjacent sleepers.

## 1. Introduction

**Figure 1:**
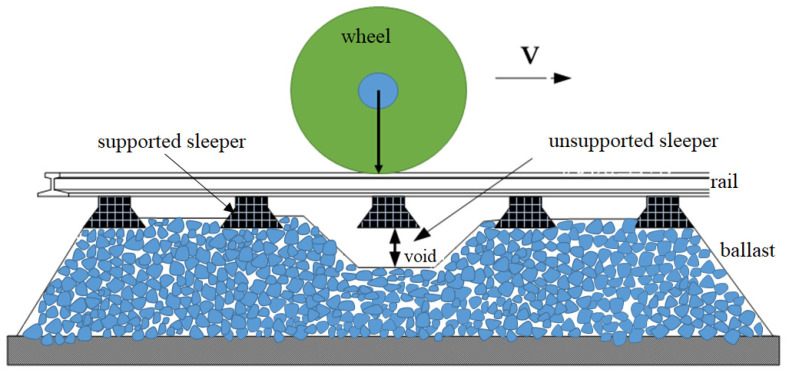
Schematic diagram of unsupported sleepers.

**Figure 2:**
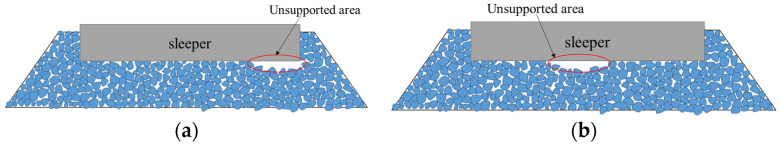
Unsupported area of sleeper: (**a**) unsupported area at the end; (**b**) unsupported area at the middle.

Ballast track is characterized by good elasticity, excellent vibration mitigation, strong water permeability, easy maintenance, fast construction, and low cost and is the most widely used structure for heavy-haul railways [1,2,3]. However, since the ballast is made of different-sized particles, it presents a non-uniform solid structure, and repeated load action will lead to wear, breaking, and displacement of the ballast particles [4]. When this uneven distribution develops to a sufficient extent, the sleeper may lose support from ballast (as depicted in Figure 1), resulting in a local unsupported sleeper. According to relevant studies, the occurrence of unsupported sleepers on ballasted track structures is relatively prevalent [5]. The sleeper may experience partial loss of support from the ballast, resulting in unsupported areas at either the middle or the end of the sleeper bottom (as shown in Figure 2). Additionally, different gaps may be observed at the two ends of a single sleeper. For the fully supported sleeper, the ballast bed is compacted to ensure optimal contact between the sleeper and particles on the top surface of the ballast bed, thereby maintaining excellent track structure integrity under load. When an unsupported sleeper is present, the compaction state of the ballast is different from its normal state, directly influencing the stability of the ballast [6,7]. In addition, the unsupported sleeper will significantly increase vibrations of the adjacent sleeper boxes on both sides and on the ballast particles below, thus accelerating the deterioration of the particles. It is therefore of great importance to study the influence of unsupported sleepers on the mechanical properties of heavy-haul railway ballast.

To date, researchers throughout the world have carried out multiple studies on unsupported sleepers: via a series of experiments and data analyses, Grassie [8] found that an unsupported sleeper increased the train load on adjacent sleepers and accelerated the rate of deterioration of the track structure. Referring to the existing literature [9,10,11,12,13,14], a coupled vehicle/track dynamic model was established to study the influence of an unsupported sleeper on the dynamic performance of vehicle and track structures; it was found that the unsupported sleeper will increase wheel/rail contact force, affect the position of the maximum bending moment of the sleeper, and aggravate the deterioration of the track structure. Ishida [4] studied the impact of loose sleepers on track dynamics damage. Wei et al. [15] established a three-dimensional transient rolling wheel/rail contact model with nonlinear ballast support using the finite element method and studied the influence of unsupported clearance and quantity on the transient wheel/rail interaction in an uneven welding area. The research results showed that a completely unsupported sleeper exacerbates the wheel/rail impact in the uneven welding area, thus increasing the yield deformation of the surface material of the rail. Zhou and Chao [16] predicted the dynamic evolution of a locally unsupported sleeper using a coupled dynamic vehicle/track model and a ballast settlement model; they found that the bearing capacity of the unsupported area decreased significantly while the load in adjacent areas increased resulting in rapid growth of the unsupported gap in adjacent areas. Zakeri [17] developed a numerical calculation model for ballasted tracks with fully supported and partially supported sleepers, investigating the influence of sleeper spacing on vertical displacement. Mosayebi [18] built a finite element model of a railway track to study the effects of the unsupported sleepers on the dynamic performance of the track and found that when a single sleeper is unsupported, the support force of adjacent sleepers is 27% higher than that of the unsupported sleeper. Cui et al. [19] studied the influence of the form and number of unsupported sleepers on the dynamic response of ballasted track using a discrete element analysis model and found that the unsupported sleeper redistributed the contact force and increased stress on the ballast particles.

To sum up, the influence of an unsupported sleeper on the dynamic performance of vehicles and tracks in most of the existing studies has been analyzed via numerical simulation, with nonlinear spring simulation of the unsupported areas used in most of the models. However, the ballast is actually a non-uniform discrete body, and the unsupported area will also affect the characteristics of ballast in adjacent areas. To date, there has been very little relevant research in this area, which may lead to inaccurate results. Therefore, in this paper, the influence of an unsupported sleeper on the vertical bearing characteristics is first analyzed using a discrete element model for the ballast. Based on this, the influence of an unsupported sleeper on the wheel/rail interaction is then analyzed at the corresponding vertical support stiffness using a coupled vehicle/track analysis model.

## 2. Discrete Element Modeling

### 2.1. Discrete Element Model of Railway Ballast

Given the discrete and discontinuous nature of railway ballast, employing the discrete element method (DEM) proves to be an effective approach for investigating its mechanical characteristics [20,21,22,23]. In an early analysis of ballast beds using this method, circular or spherical elements are commonly utilized to simulate the ballast particles in 2D or 3D, respectively. By adjusting the inter-particle contact parameters, it is possible to make their macroscopic mechanical properties align with those observed in real-world ballast [24,25]. However, numerous experiments and simulation calculations have demonstrated that the surface particle size distribution and geometric shape significantly influence the mechanical performance of ballast. The irregular shape of ballast particles enables them to interlock with each other, which has a significant impact on simulating the mechanical behavior of railway ballast beds. In order to accurately simulate ballast angularity, a DEM model was established based on the actual shape of the ballast particles. For this study, 10 ballast particles with different shapes were selected for laser scanning by creating three-dimensional surface models. These models were then imported into the discrete element software PFC 6.0 and filled with spherical units to form clumps, as shown in Table 1; for experimental purposes, this is sufficiently close to the real shape of the ballast. The shapes of certain ballast particles are shown in Table 1. The distribution of ballast particles is uniform across all shapes.

**Figure 3:**
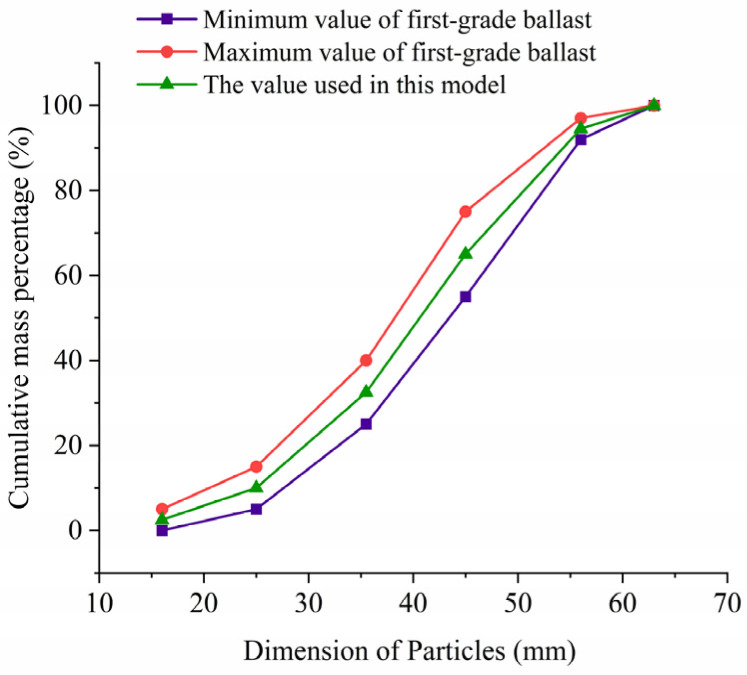
Grading curve of ballast.

According to the requirements of Chinese standards “Railway Ballast” (TB/T2140-2008 [26]) and the “Code for Design of Heavy Haul Railways” (TB10625-2017 [27]), the first-grade ballast (as shown in Figure 3) for new railways is used to create ballast particles across all shapes.

**Figure 4:**
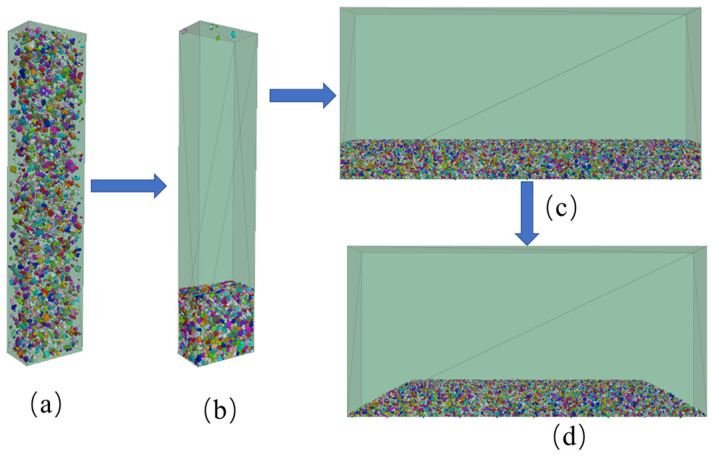
Process of DEM ballast model establishment: (**a**) ballast particles distributed in the box; (**b**) initial stacking equilibrium state of the ballast particles; (**c**) ballast particles assembled in the ballast box; (**d**) form the ballast bed.

**Figure 5:**
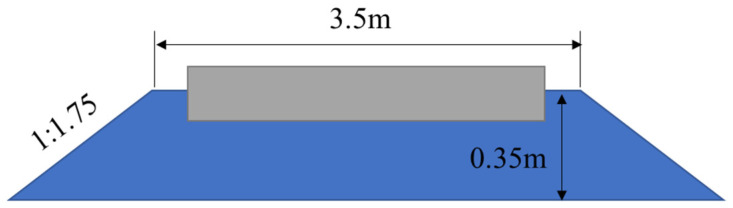
Ballast bed cross section.

The DEM ballast model was developed by initially creating a box filled with first-grade ballast particles, which had a porosity of 98%. The height of the box significantly exceeds that of the ballast (as depicted in Figure 4a). Secondly, a gravitational force of 100 times the normal gravity is applied to the model in order to achieve the initial stacking equilibrium state of the ballast particles (as shown in Figure 4b). Subsequently, a wall with a servo mechanism is employed to compact the ballast particles, after which a large contact force between the ballast particles is released via servo unloading. Afterward, the ballast particles within the box are replicated and assembled in a ballast box (as illustrated in Figure 4c). Finally, the excess ballast particles are removed to form the actual ballast bed (as demonstrated in Figure 4d). The sectional form of the ballast bed meets the design requirements for heavy-haul railway stipulated in the “Code for Design of Railway Track” (TB10082-2017 [28]): The width of the top surface of the ballast is 3.5 m, the height of the ballast shoulder is 150 mm, the ballast thickness is 0.35 m, and the ballast slope is 1:1.75 (as shown in Figure 5).

**Figure 6:**
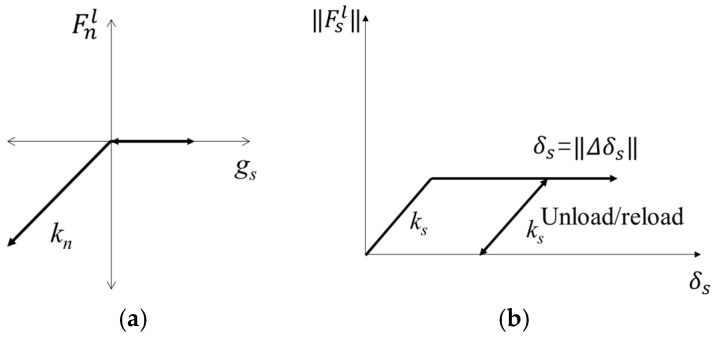
Force/displacement law for the linear component of the contact model: (**a**) normal force versus surface gap; (**b**) shear force versus relative shear displacement.

In this model, the deformation and breaking of the ballast particles were not considered. Each ballast particle was modeled as a clump consisting of rigid assemblies of spherical pebbles. These clumps possessed the ability to translate and rotate, with their motion governed by the equations of motion. The mass, centroid position, and inertia tensor can be computed based on the geometric characteristics and density of the object. In this model, the interactions between the particles and those between particles with the walls of the ballast box are simplified as a linear contact model. The linear model comprises a linear and a dashpot component that acts in parallel with one another. The linear component provides linear elastic (no-tension) frictional behavior, while the dashpot component provides viscous behavior. Both components act over a vanishingly small area and thus transmit only a force. The linear force is produced by linear springs with constant normal and shear stiffnesses, *k_n_* and *k_s_*. The force/displacement law for the linear contact model is illustrated in Figure 6. $F_{n}^{l}$ and $F_{s}^{l}$ are the normal and shear components of the linear contact force. $g_{s}$ is the surface gap, which is defined as the difference between the contact gap and the reference gap. $\delta_{s}$ is the relative shear displacement between two contact pieces.

The main parameters of the DEM ballast model used are shown in Table 2.

**Figure 7:**
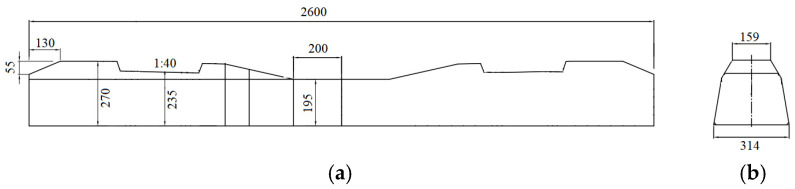
Schematic diagram of Chinese Type IV concrete sleeper: (**a**) front view; (**b**) side view.

**Figure 8:**
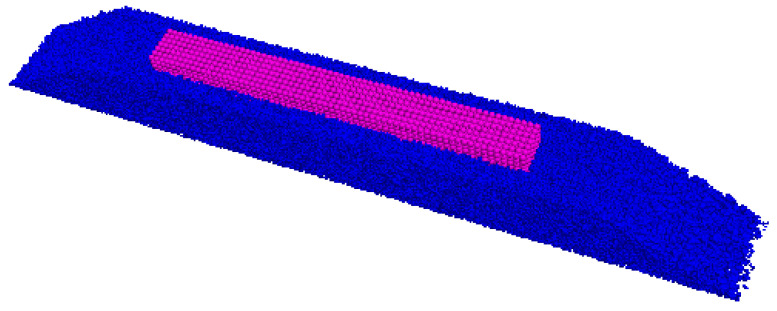
Diagram of the ballast model.

In the discrete element model, continuous media such as sleepers can be simulated as a wall or clump. The wall can accurately reflect the true shape of the sleeper and accurately determine the number of contacts between the sleeper and the ballast, allowing the contact force between the sleeper and the ballast to be reflected more accurately. However, since the wall itself has no mass property in the discrete element model and will not move under the action of an external force, a servo mechanism must be introduced to simulate the dead weight of the sleeper. For the servo mechanism, the load is usually applied in the form of velocity and displacement, with its value determined by the contact force against the wall. This method usually incurs a significant error due to the indirect application of the load on the wall. In the simulation of the sleeper using clump, the sleeper profile is filled with rigid spheres, with particles of corresponding density used to simulate the dead weight of the sleeper. However, it is difficult to accurately obtain the number of contacts between the sleeper and the ballast using this method. Increasing the number of spheres filled in the polymer can reduce the error of the contact number. Therefore, in this paper, a clump is used to simulate a Chinese Type IV concrete sleeper (as shown in Figure 7) on a heavy-haul railway ballast track. The sleep clump consists of rigid spheres arranged in a regular pattern, with no relative displacement between spheres. The clump exhibits both translational and rotational motion without any elastic deformation. The mass, centroid position, and inertia tensor can be computed based on the g spheres and density. The sleeper is embedded into the track bed, with the top surface of the ballast bed positioned 3 cm lower than that of the sleeper. Considering the calculation efficiency of discrete elements, only one sleeper is considered in the ballast model, as shown in Figure 8.

### 2.2. Verification of Ballast Model

**Figure 9:**
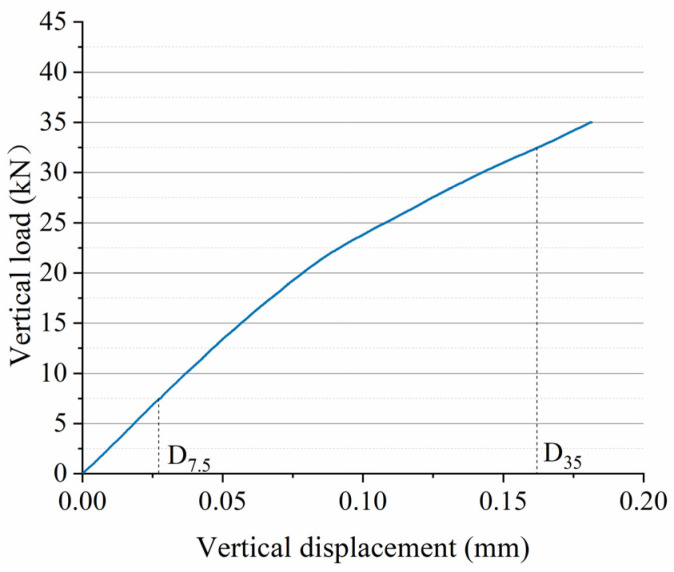
Vertical displacement/load curve of the fully supported sleeper simulated by the dem ballast model.

The vertical displacement/load curve of the fully supported sleeper simulated by the DEM ballast model was shown in Figure 9. From Figure 9, we can find that the vertical displacement of the sleeper gradually increased with the vertical load; the trend is in accordance with the practical displacement change of the sleeper.

Based on the “code for Chinese jointless track design”, the vertical forces of 7.5 kN and 35 kN are applied to the middle of the sleeper. After it has stabilized, the corresponding vertical displacements *D*_7.5_ and *D*_35_ (as shown in Figure 9) are extracted to calculate the bearing stiffness *k_s_* by the following equation:

(1) $$k_{s}=\frac{27.5}{D_{35}-D_{7.5}}$$

**Figure 10:**
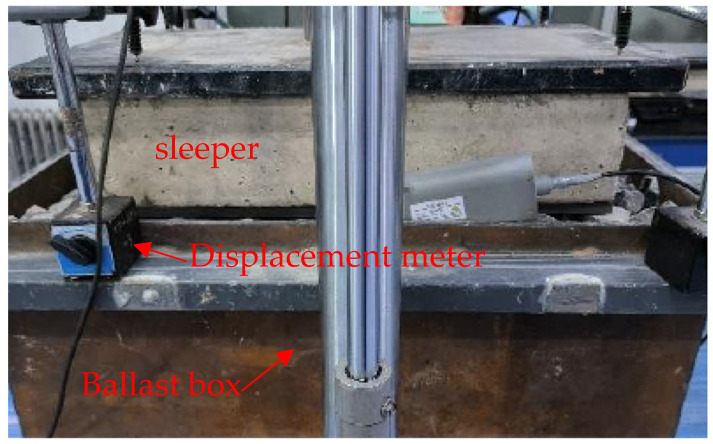
Experiment of ballast vertical bearing stiffness.

Han [29] utilized a ballast box to test the vertical bearing stiffness of ballast, as shown in Figure 10. To achieve compaction, he applied 5000 cycles of loading with a frequency of 3 Hz and an amplitude ranging from 1 kN to 28 kN. Subsequently, a vertical load was applied to test the ballast bearing stiffness.

The bearing stiffness obtained from simulation and experimentation are presented in Table 3. The results showed that the experimental bearing stiffness is measured as 184 kN/mm, while the simulated bearing stiffness is 173.63 kN/mm; the difference is only 6.1%. The experiment and the simulation are highly consistent, which indicates that the DEM model in this study is able to reflect the bearing characteristics of ballast.

## 3. Bearing Characteristics of Ballast Bed with an Unsupported Area

**Figure 11:**
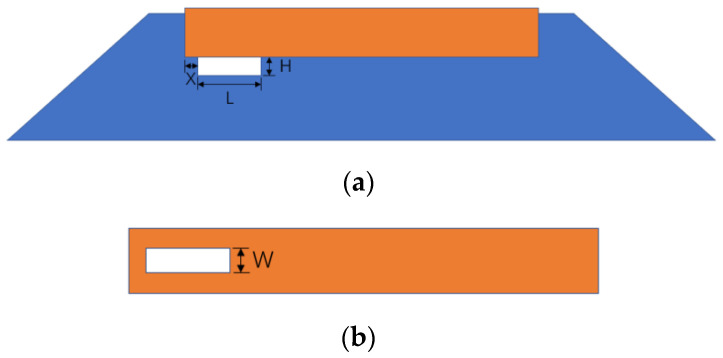
Diagram of unsupported ballast: (**a**) cross section; (**b**) top view.

As shown in Figure 11, the unsupported area may be located at the middle or the end of the sleeper bottom. According to previous research, compared with the middle unsupported area, a unilaterally unsupported sleeper (as depicted in Figure 11b) poses a greater threat to the long-term operating safety of trains. For this reason, this paper primarily focuses on the study of unilaterally unsupported sleepers. The unsupported area is simulated by removing ballast particles that come into contact with the bottom of the sleeper. Considering the influence of the length (L), height (H), and width (W) of an unsupported sleeper on bearing characteristics, as well as the displacement (X) from the end of the sleeper, the diagram for the unsupported sleeper is shown in Figure 3.

### 3.1. Distribution of Contact Force

**Figure 12:**
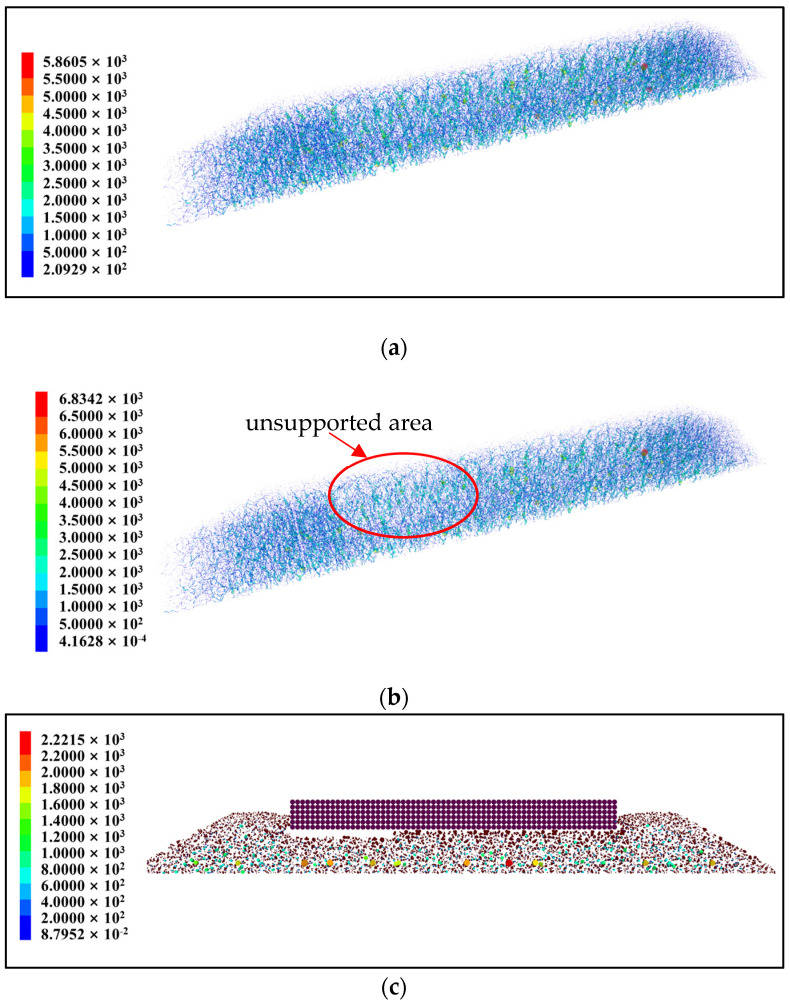
Contact force chain of ballast beds: (**a**) contact force chain of an intact ballast bed; (**b**) contact force chain of an unsupported ballast bed; (**c**) cross section of unsupported ballast bed contact force chain, the unit is N.

The contact force on the ballast bed is an important factor affecting its stability. Figure 12a shows the contact force chain of an intact ballast bed, while Figure 12b shows the contact force chain on a ballast bed with an unsupported length, height, and width of 800 mm, 60 mm, and 280 mm, respectively, with a displacement from the sleeper end of 0 mm. It can be clearly seen from the figures that there is a uniform contact force distribution of intact ballast for a vertical load and that the force chain is relatively complete within the scope of the ballast bed. For the unsupported sleeper, although the ballast particles beneath the unsupported area do not have direct contact with the sleeper, except for the topmost particles, a significant portion of them actively contribute to bearing the vertical force transmitted from the sleeper, as depicted in Figure 12c. The ballast bed also serves as a whole to withstand vertical force, exhibiting a continuous and uniform chain of contact force, as shown in Figure 12b. However, the topmost ballast particles on the unsupported area fail to bear the vertical load effectively, leading to a significant increase in contact force among all ballast particles. At a vertical load of 35 kN, the maximum contact force of the intact ballast bed is 5.86 kN, while the maximum contact force of the ballast bed with the unsupported sleeper increases to 6.843 kN, representing an increase of 16.7%. It can therefore be seen that the unsupported sleeper will accelerate the deterioration of ballast particles.

### 3.2. Influence of Unsupported Length on Vertical Bearing Stiffness

**Figure 13:**
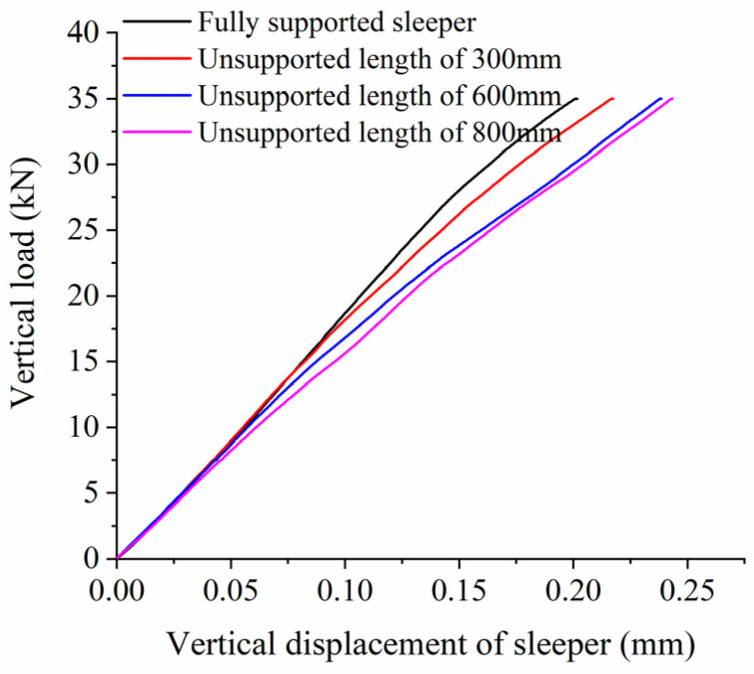
Vertical load and displacement of sleeper at different unsupported lengths.

**Figure 14:**
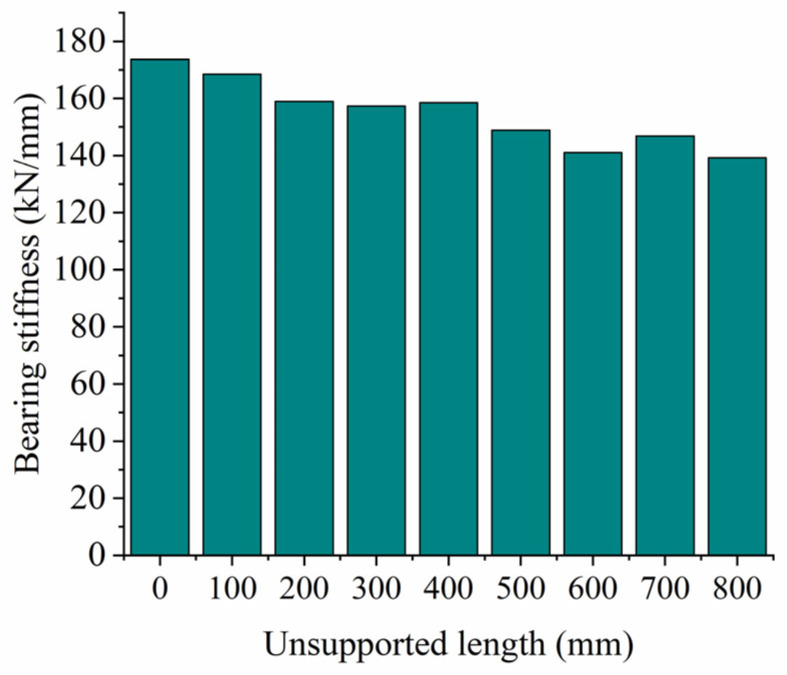
Vertical bearing stiffness at different unsupported lengths.

When analyzing the influence of the unsupported length on vertical bearing stiffness, we assume an unsupported height H = 0.06 m, a displacement from the sleeper end X = 0 m, and an unsupported width W = 0.28 m; in other words, the sleeper is completely unsupported longitudinally along the track. Figure 13 shows the relationship between vertical displacement and force on the sleeper for different unsupported lengths, while Figure 5 shows the vertical bearing stiffness of the ballast bed for the same conditions. As can be seen from Figure 13, when the vertical load is less than 5 kN, there is little influence from the unsupported sleeper on the vertical displacement of the sleeper, and the displacement and load curves more or less coincide with each other. The vertical displacement of the sleeper under the same vertical load then gradually increases as the unsupported length increases. The displacement and load curves of the fully supported sleeper start out being linear, and then gradually begin to show nonlinear characteristics as the load increases while the displacement slows down. On an unsupported sleeper, the length of the linear stage changes; when the unsupported length reaches 800 mm, it displays largely linear characteristics across the whole loading stage. The vertical displacement of the sleeper continues to increase linearly with increasing load, with a displacement that is significantly larger than that of the fully supported sleeper. As can be seen from Figure 14, as the unsupported length increases, the supporting area of the sleeper decreases accordingly, as does the vertical bearing stiffness of the ballast bed, while the reduction ratio gradually increases. The vertical bearing stiffness of the intact ballast bed is 173.63 kN/mm; for an unsupported length of 300 mm, the vertical bearing stiffness is 157.29 kN/mm, a decrease of 9.4% compared with the intact ballast bed. For an unsupported length of 800 mm, the vertical bearing stiffness is 139.23 kN/mm, a decrease of 19.8% compared to the intact ballast bed.

### 3.3. Influence of Unsupported Width on Vertical Bearing Stiffness

When analyzing the influence of the unsupported width on the vertical bearing stiffness, we assume an unsupported length L = 0.8 m, an unsupported width H = 0.06 m, and a displacement from the sleeper end of X = 0 m.

**Figure 15:**
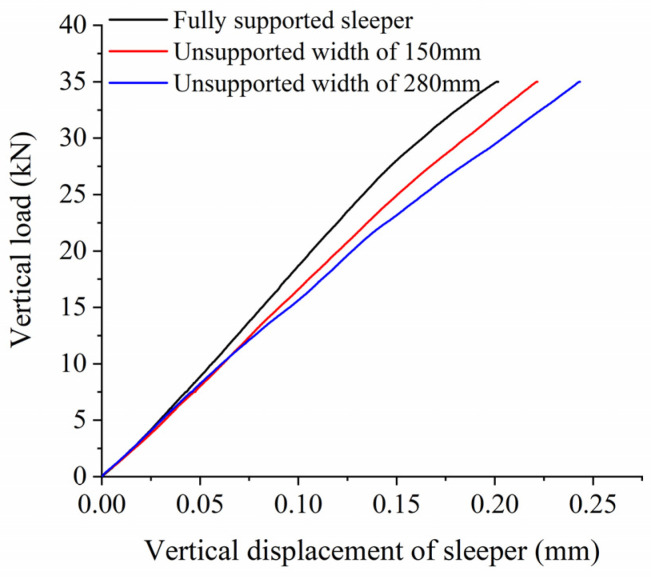
Vertical load and displacement of sleeper at different unsupported widths.

**Figure 16:**
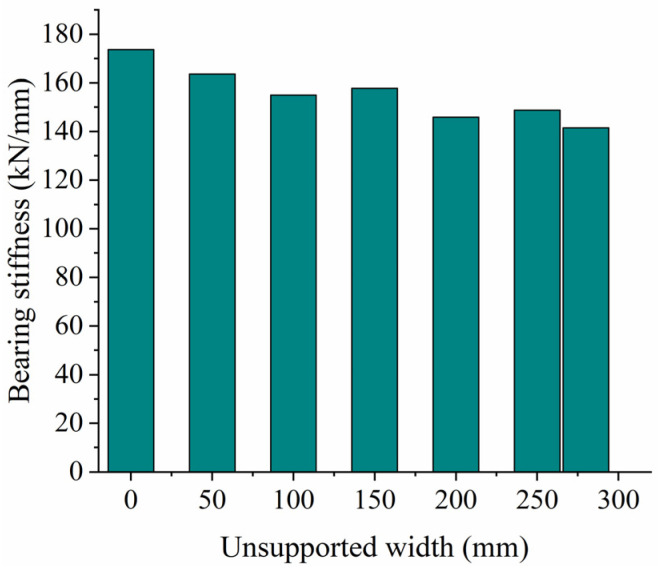
Vertical bearing stiffness at different unsupported widths.

Figure 15 shows the relationship between vertical displacement and force on the sleeper for different unsupported widths, while Figure 16 shows the vertical bearing stiffness of the ballast bed for the same conditions. As can be seen from Figure 15, when the vertical load is less than 7.5 kN, the vertical displacement of the sleeper basically remains the same for different unsupported widths. This vertical displacement then increases gradually as the unsupported width increases, and the linear stage becomes longer during the loading test of the ballast bed. As can be seen from Figure 16, as the unsupported width increases, the vertical bearing stiffness of the ballast bed gradually decreases. When the sleeper is completely unsupported in the width direction, the vertical bearing stiffness of the ballast bed decreases from 173.63 kN/mm (that of an intact ballast bed) to 141.46 kN/mm, a decrease of 18.5%.

### 3.4. Influence of Unsupported Height on Vertical Bearing Stiffness

**Figure 17:**
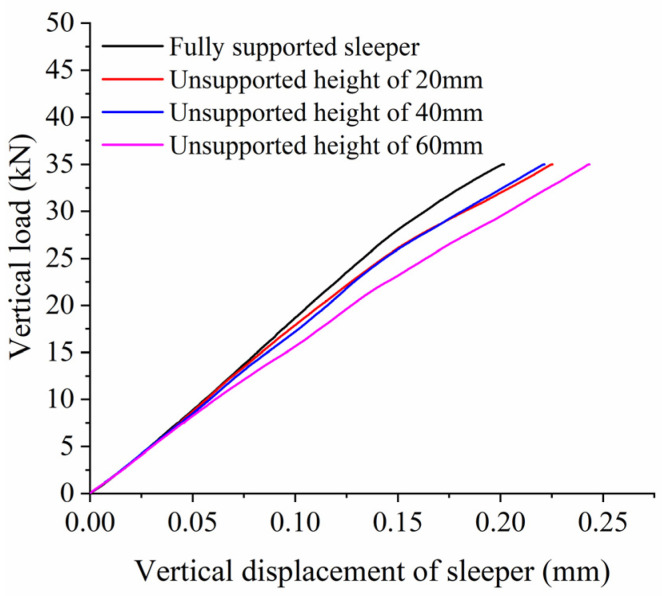
Vertical load and displacement of sleeper at different unsupported heights.

**Figure 18:**
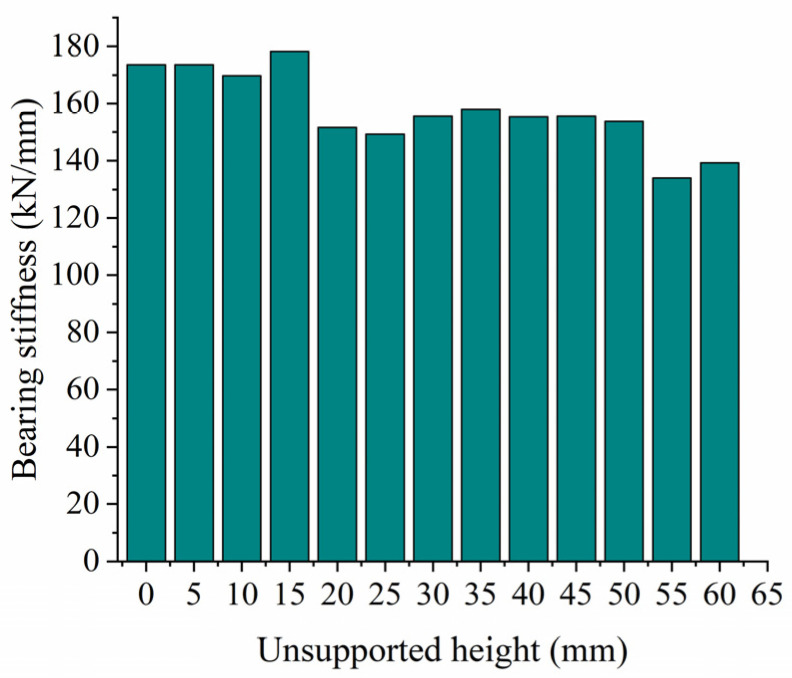
Vertical bearing stiffness at different unsupported heights.

When analyzing the influence of the unsupported height on vertical bearing stiffness, we assume an unsupported length L = 0.08 m, a displacement from the sleeper end X = 0 m, and an unsupported width W = 0.28 m. Figure 17 shows the relationship between vertical displacement and force on the sleeper for different unsupported heights, while Figure 18 shows the vertical bearing stiffness of the ballast bed for the same conditions. As can be seen from the figures, when the unsupported height is less than 15 mm, the vertical bearing stiffness of the ballast bed remains almost unchanged compared to that of the intact ballast bed. As the unsupported height increases to 20 mm, the vertical bearing stiffness of the ballast bed significantly decreases from 173.63 kN/mm (that of an intact ballast bed) to 151.66 kN/mm, a decrease of 12.6%. After that, when the unsupported height is within the range of 20 mm~50 mm, the vertical bearing stiffness of the ballast bed changes very little, and the force and displacement curves for different unsupported heights more or less coincide with each other, indicating that a change in unsupported height will not cause any significant change in vertical bearing stiffness of the ballast bed within this range. However, once the unsupported height becomes greater than 50 mm, the vertical bearing stiffness decreases sharply: when the height is increased from 50 mm to 55 mm, it decreases from 153.79 kN/mm to 134.23 kN/mm, a reduction of 12.8%.

### 3.5. Influence of Unsupported Position on Vertical Bearing Stiffness

**Figure 19:**
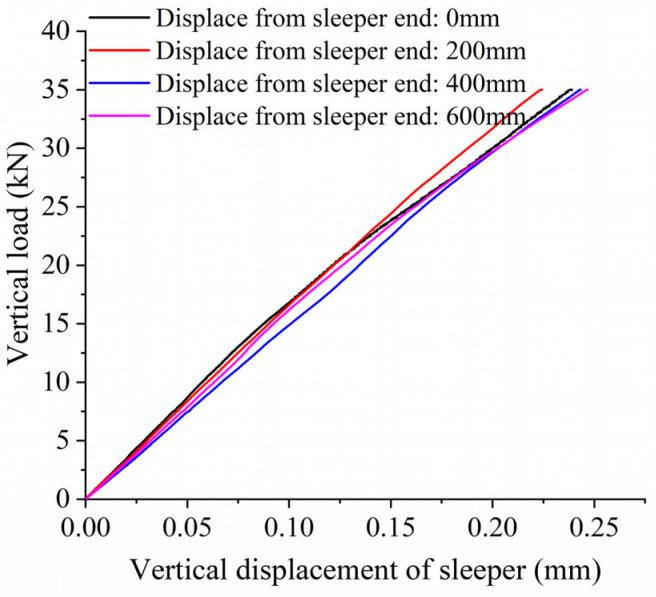
Vertical load and displacement of sleeper with unsupported area at different positions.

**Figure 20:**
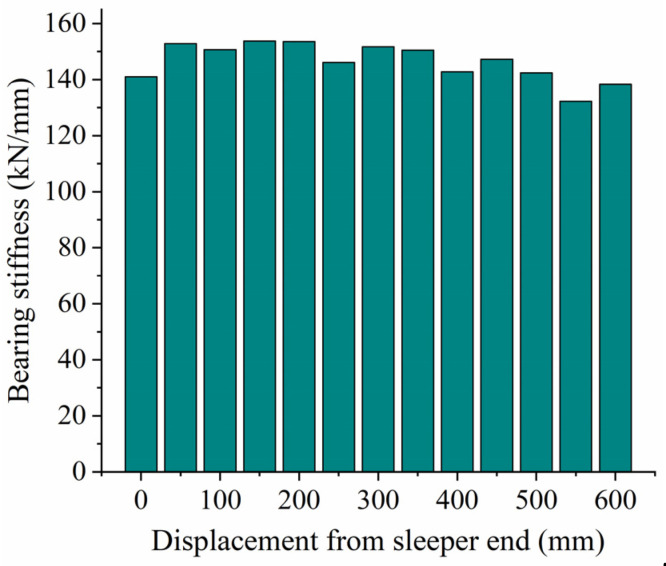
Vertical bearing stiffness of ballast with unsupported area at different positions.

When analyzing the influence of unsupported position on vertical bearing stiffness, we assume an unsupported length L = 0.06 mm, an unsupported width W = 0.28 m, and an unsupported height H = 0.06 m. Figure 19 shows the relationship between vertical displacement and force on the sleeper for different unsupported positions, while Figure 20 shows the vertical bearing stiffness of the ballast bed for the same conditions. As can be seen from the figures, the vertical stiffness of the ballast bed will change at different unsupported positions as unsupported area and height change. When the distance from the unsupported sleeper to the sleeper end is less than 200 mm, the vertical bearing stiffness of the ballast bed increases slightly as the unsupported position continues to move closer to the center of the sleeper. When the displacement from the sleeper end increases from 0 mm to 200 mm, the vertical bearing stiffness increases from 139.23 kN/mm to 153.6 kN/mm, an increase of 10.3%. When the displacement from the sleeper end is greater than 300 mm, the vertical bearing stiffness decreases gradually as the unsupported position moves closer to the center of the sleeper; when the displacement from the sleeper end is 600 mm, the vertical bearing stiffness decreases to 138.34 kN/mm.

## 4. Influence of an Unsupported Sleeper on Wheel/Rail Interaction

### 4.1. Vehicle/Track Coupling Dynamics Model

When looking at the discrete element analysis results for the ballast bed, due to the discrete structure of the track bed, a locally unsupported sleeper will not only cause the sleeper to no longer play a supporting role but will also cause a change in the vertical support performance of the entire sleeper. This, in turn, will cause its vertical bearing stiffness to decrease. Therefore, based on the vehicle/track coupling dynamics theory [30], a vehicle/track coupling dynamics model, including the train subsystem and track subsystem, is established to study the influence of the unsupported area of a sleeper on the wheel/rail dynamical interaction.

**Figure 21:**
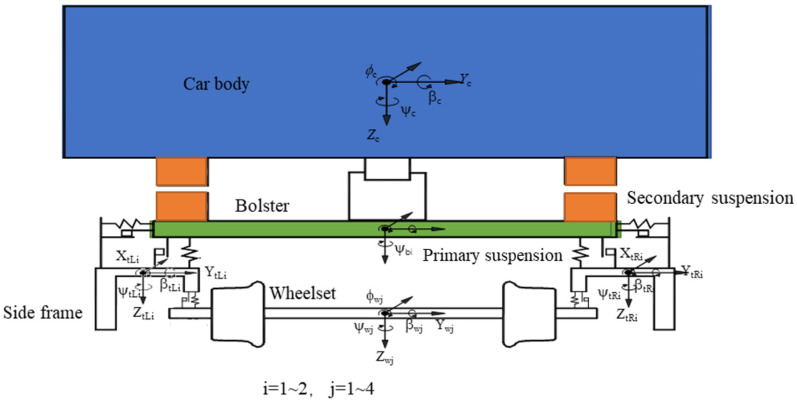
Front view of vehicle subsystem.

The vehicle system is simulated using a C80 truck dynamics model with a 25 t axle load, incorporating both the first and second series suspension, as shown in Figure 21. In this dynamic model, the wheelset, side frame, bolster, and car body are both regarded as rigid bodies. The degrees of freedom associated with the structural configuration of this vehicle subsystem are presented in Figure 13, encompassing a total of 47 degrees of freedom.

The equation of motion of the freight vehicle subsystem is derived according to D’Alembert’s principle, which is the second-order ordinary differential equation expressed in the matrix form as follows:

(2) $$M\overset{\text{..}}{X}=F_{n}+F_{t}+F_{w}+F_{g}+F_{d}+F_{s}+F_{f}$$

where *M* is the system mass matrix; *F_n_* represents the normal wheel/rail contact force vector; *F_t_* is the tangential wheel/rail contact force vector; *F_w_* is the gravity vector; *F_g_* denotes the gyroscopic force vector; *F_d_* is the damping force vector; *F_s_* denotes the spring force vector; *F_f_* is the friction force vector.

**Figure 22:**
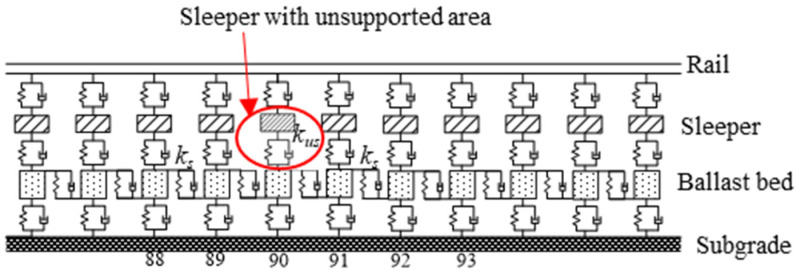
Track subsystem.

In the ballasted track structure, all components, including the rail, sleeper, and track bed, actively contribute to vibration phenomena (as illustrated in Figure 22). The rail is represented by a finite-length Euler beam model, capable of exhibiting vibrations in both the vertical and transverse direction as well as twist deformation. The sleeper is treated as a rigid body, accounting for its vertical and transverse displacements as well as rotational degrees of freedom. The rail and sleeper, the sleeper and track bed, and the track bed and subgrade are interconnected via linear springs and viscous damping. The ballast bed is divided into discrete mass blocks based on the actual sleeper spacing, with a sole focus on the vertical vibration.

The equation of the track subsystem is expressed as

(3) $$M_{t}{\overset{\text{..}}{u}}_{t}+K_{t}u_{t}+C_{t}{\dot{u}}_{t}=F_{t}$$

where *M_t_*, *K_t_*, and *C_t_* are mass, stiffness, and damper matrices of the track subsystem, respectively; *u_t_*, ${\dot{u}}_{t}$ , and ${\overset{\text{..}}{u}}_{t}$ are displacement, velocity, and acceleration vectors of the track subsystem, respectively; *F_t_* is the force vector.

According to the aforementioned research, it is evident that although there is no direct contact between the sleeper and the ballast in the unsupported area, the ballast beneath the unsupported area actively contributes to bearing the vertical load transmitted from the sleeper. Consequently, conventional nonlinear spring models are deemed inadequate for simulating unsupported sleepers. Therefore, the vertical bearing stiffness of the ballast bed obtained in the discrete element model (*k_us_*) is used to fully replace the full bearing stiffness (*k_s_*) of a completely unsupported sleeper for this study. In this way, the influence of different unsupported heights and lengths on wheel/rail interaction can be analyzed. The main parameters of this dynamical model are shown in Table 4.

### 4.2. Vertical Wheel/Rail Force

(1) Influence of Unsupported Length

**Figure 23:**
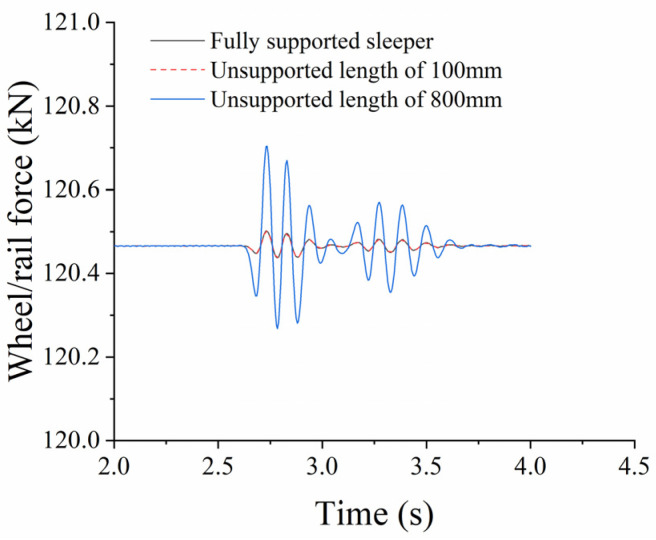
Time-based wheel/rail force against unsupported length.

**Figure 24:**
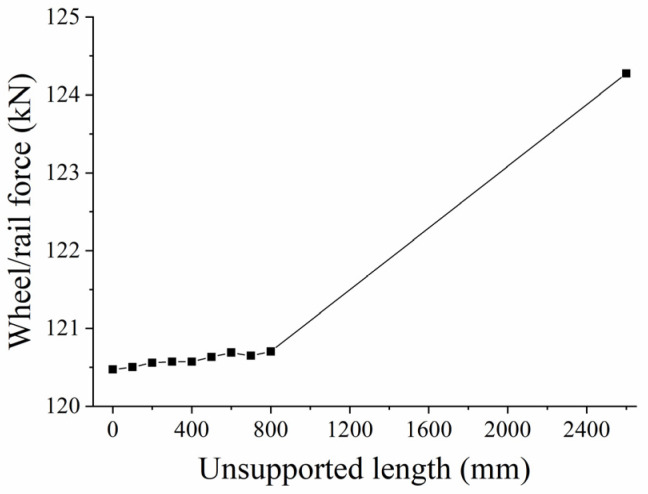
Wheel/rail force change against unsupported length.

When the unsupported height H = 0.06 m, the displacement from the sleeper end X = 0 m, and the unsupported width W = 0.28 m. The longitudinal wheel/rail force changes with time, as shown in Figure 23, while the change in force against unsupported length is shown in Figure 24. It can be seen from the figures that the unsupported sleeper will cause an unstable stiffness within the track structure, which will, in turn, lead to a dynamic change in the wheel/rail interaction force. The maximum longitudinal wheel/rail force on the fully supported sleeper is 120.472 kN. As the unsupported length increases, the maximum longitudinal wheel/rail force also increases. When the length reaches 800 mm, the maximum longitudinal wheel/rail force is 120.704 kN, representing an increase of 0.19%. However, when the unsupported length increases to 2600 mm, which represents a fully unsupported sleeper, the maximum longitudinal wheel/rail force is 124.276 kN, an increase of 3.15% over that of an intact ballast bed (with an unsupported height of 0.06).

(2) Influence of Unsupported Height

**Figure 25:**
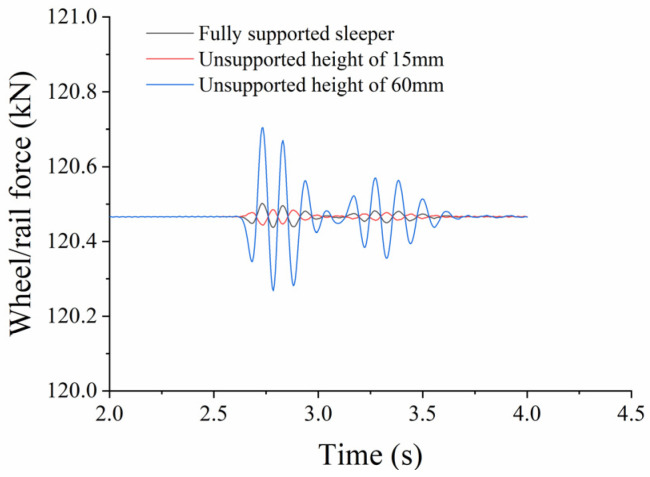
Time-based longitudinal wheel/rail force against unsupported height.

**Figure 26:**
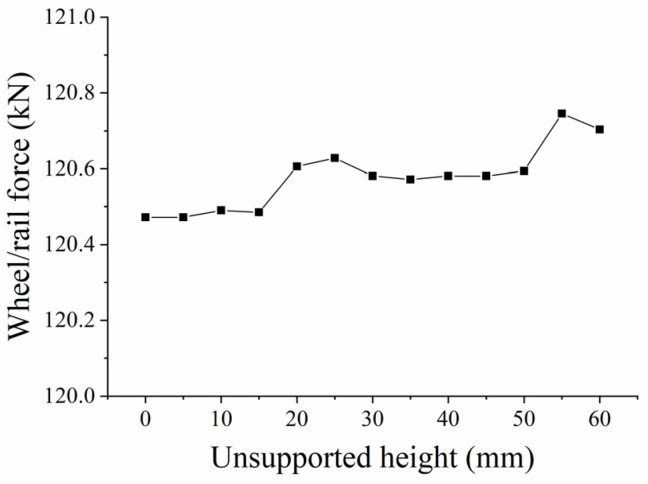
Longitudinal wheel/rail force against unsupported height.

When analyzing the influence of the unsupported height on wheel/rail power interaction, we assume an unsupported length L = 0.08 m, a displacement from the sleeper end X = 0 m, and an unsupported width W = 0.28 m. Figure 25 shows the change in wheel/rail force with time for different unsupported heights, while Figure 26 shows the change in maximum longitudinal wheel/rail force against unsupported height. It can be seen from the figures that the longitudinal wheel/rail force is basically the same when the unsupported height is less than 15 mm. When it reaches 20 mm, the longitudinal wheel/rail force increases slightly, and as height continues to increase up to 50 mm, the longitudinal wheel/rail force remains basically the same. It then increases linearly as the height increases beyond 50 mm. During the stage where the unsupported height increases from 0 to 60 mm, the maximum longitudinal wheel/rail force only increases from 120.261461 kN to 120.704 kN, representing an increase of 0.19%.

### 4.3. Sleeper Supporting Force

(1) Influence of Unsupported Length

**Figure 27:**
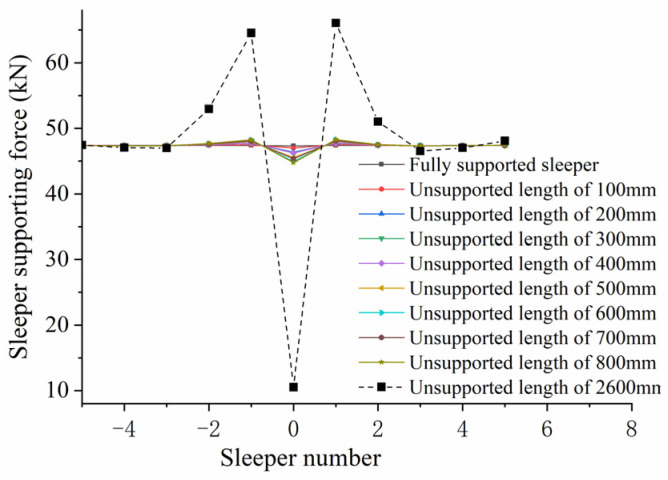
Sleeper supporting force against unsupported length.

Figure 27 shows the change in sleeper supporting force against unsupported length. The horizontal coordinates indicate sleeper positions: “−5” indicates the fifth sleeper on the left side of the unsupported sleeper, while “+5” indicates the fifth sleeper on the right side of the unsupported sleeper. It can be seen from the figure that the supporting force of the unsupported sleeper decreases as the train passes through while the sleeper supporting force on both sides increases. This occurs because the sleeper in the track structure is not independent but consists of a section of track made of rails and fastening systems connected in series to jointly bear the train load. When there is an unsupported sleeper present, the supporting force of this sleeper is weakened, meaning it can only bear a smaller train load; this reduced load then has to be borne by the sleepers on either side. As can be seen from the figure, the unsupported sleeper causes an increase in the supporting force on the two sleepers on either side of the unsupported area. The longer the unsupported length, the smaller the supporting force on the unsupported sleeper, and the greater the supporting force on the sleepers on either side. When the unsupported sleeper length increases from 0 to 800 mm, the supporting force on the first sleeper on the left increases from 47.413 kN to 48.272 kN, an increase of 1.8%; in the same period, the supporting force of the first sleeper on the right increases from 47.413 kN to 48.314 kN, an increase of 1.9%. The changes in supporting force on the left and right sides of the sleeper remain more or less the same. When the whole sleeper is completely unsupported, the supporting force on the unsupported sleeper decreases sharply to 10.498 kN, a decrease of 77.8% from that of a fully supported sleeper; the supporting force on the first sleeper on the left increases to 64.569 kN, an increase of 26.5%. The supporting force of the second sleeper on the left increases to 52.999 kN, an increase of 11.8% compared to that of the fully supported sleeper.

(2) Influence of Unsupported Height

**Figure 28:**
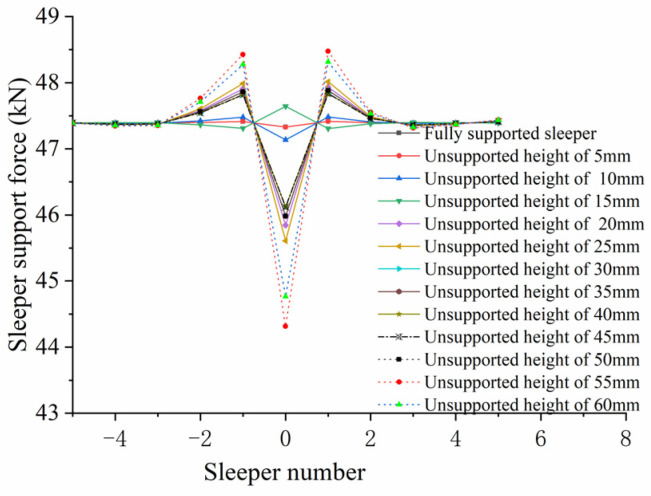
Sleeper supporting force against unsupported height.

Figure 28 shows how the sleeper’s supporting force varies with the unsupported height. As can be seen from the figure that when the unsupported height is less than 15 mm, it has little influence on the sleeper supporting force; when the unsupported height lies in the range of 20~50 mm, the sleeper supporting force in the unsupported area is less than the supporting force of the fully supported sleeper, while the supporting forces of the sleepers on either side are greater than that of the fully supported sleeper. However, the change in unsupported height in this range will not cause any significant change in sleeper supporting force. When the unsupported height is greater than 50 mm, the supporting force of the unsupported sleeper decreases as the unsupported height increases, while the supporting forces of the sleepers on both sides also increase. When the unsupported height is 60 mm, the supporting force of the unsupported sleeper is reduced from 47.327 kN to 44.767 kN, 5.4% less than for a fully supported sleeper; the supporting force of the first sleeper on the left increases from 47.413 kN to 48.217 kN, an increase of 1.8%.

## 5. Conclusions

In this paper, a three-dimensional discrete element model was established to analyze the influence of an unsupported sleeper on the vertical bearing characteristics of the ballast bed, and the bearing stiffness was then applied to a dynamic vehicle/track coupling model to study the influence of an unsupported sleeper on the wheel/rail interaction. The following conclusions were drawn:

(1) For the unsupported sleeper, although the ballast particles beneath the unsupported area do not have direct contact with the sleeper, except for the topmost particles, a significant portion of them actively contribute to bearing the vertical force transmitted from the sleeper. Consequently, conventional nonlinear spring models are deemed inadequate for simulating unsupported sleepers.
(2) An unsupported sleeper will reduce both the supporting area of the ballast bed under the sleeper, and the number of ballast particles, thus increasing the contact force between the particles and accelerating the deterioration of ballast particles.
(3) When the vertical load is less than 5 kN, there is little influence from the unsupported length on the vertical displacement. As the unsupported length increases, as does the vertical bearing stiffness of the ballast bed decreases accordingly. For an unsupported length of 800 mm, the vertical bearing stiffness is 139.23 kN/mm, representing a decrease of 19.8% compared to the intact ballast bed.
(4) When the vertical load is less than 7.5 kN, the vertical displacement of the sleeper basically remains the same for different unsupported widths. As the unsupported width increases, the vertical bearing stiffness of the ballast bed gradually decreases. When the sleeper is completely unsupported in the width direction, the vertical bearing stiffness of the ballast bed decreases from 173.63 kN/mm (that of an intact ballast bed) to 141.46 kN/mm, representing a decrease of 18.5%.
(5) When the unsupported height is less than 15 mm, there is little influence on the vertical bearing stiffness of the ballast bed. In the range of 20~50 mm, the vertical bearing stiffness of the ballast bed is significantly reduced compared with that for the intact ballast but does not vary as height increases within this range. When the height exceeds 50 mm, the vertical bearing stiffness decreases significantly as height increases. When the height is increased from 50 mm to 55 mm, it decreases from 153.79 kN/mm to 134.23 kN/mm, a reduction of 12.8%.
(6) Different unsupported positions also have different influences on the vertical bearing stiffness of the ballast bed: when the sleeper ends are unsupported and the unsupported distance is greater than 500 mm, the vertical bearing stiffness of the ballast bed remains relatively small.
(7) When a single sleeper is unsupported, even though an increase in the unsupported length and height will cause a corresponding increase in longitudinal wheel/rail force to different extents, the actual change is very small. Therefore, a single unsupported sleeper has limited influence on wheel/rail interactions.
(8) An increase in unsupported length and height will lead to a decrease in the sleeper supporting force exerted on the unsupported position and to an increase in the supporting force exerted by the sleepers on the left and right sides of the unsupported sleeper. However, a partially unsupported sleeper at one end has little influence on the overall sleeper supporting force, and the associated change in amplitude remains small. When a single sleeper is completely unsupported, the sleeper supporting force will change significantly, which will, in turn, accelerate the deterioration of the ballast bed.

It can therefore be concluded that the unsupported sleeper significantly affects the overall performance of the ballast bed and its vertical stiffness. Consequently, it would accelerate the deterioration of ballast particles. Hence, once a sleeper becomes unsupported, it must be repaired as soon as possible.

It is worth noting that there are still several areas for improvement in this paper. The sleeper is simulated as a clump in the DEM model of ballast, which inaccurately simulates its contact with ballast particles and fails to reflect the influence of the sleeper’s elastic deformation on the model. Therefore, it is necessary to develop a finite element-discrete element coupling model in future research to obtain more accurate calculation results. In terms of vehicle/track coupling dynamics analysis, this paper primarily focuses on the impact of a single unsupported sleeper on both the sleeper supporting force and wheel/rail dynamics. However, in practical engineering scenarios, multiple sleepers with unsupported areas commonly occur. Hence, it is meaningful to analyze the influence of multiple unsupported sleepers on the dynamic characteristics of the wheel/rail system and track system in future studies.

## Figures and Tables

**Table 1:** Three-dimensional surface model of ballast particles.

No.	1	2	3	4	5
Shape	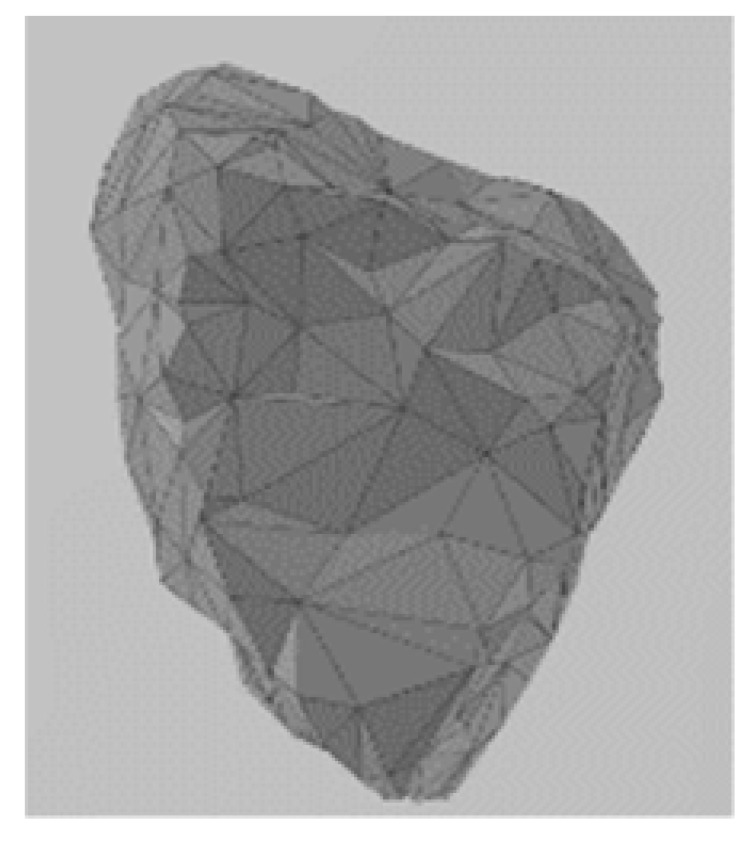	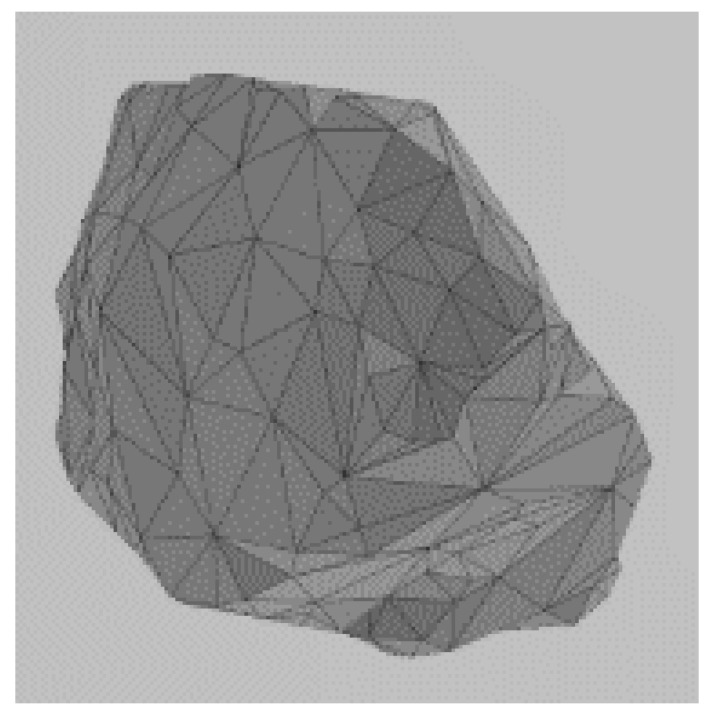	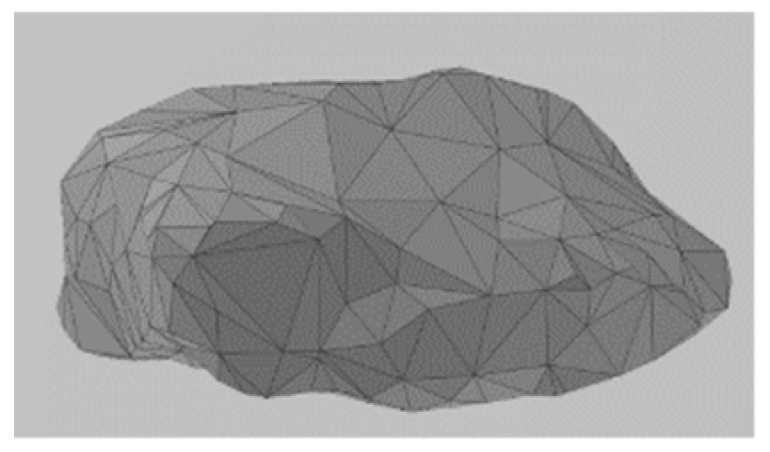	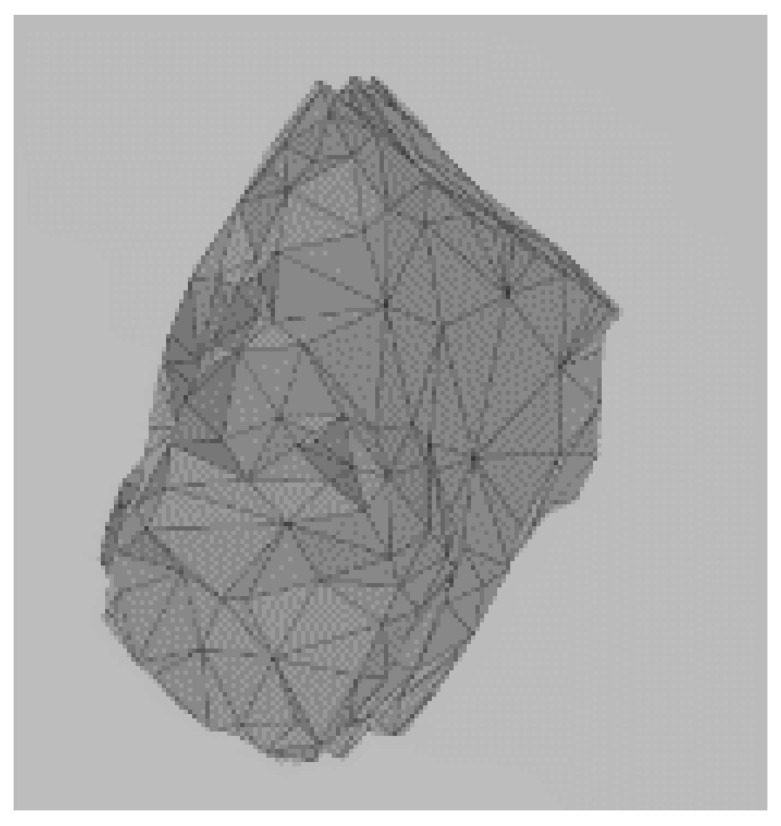	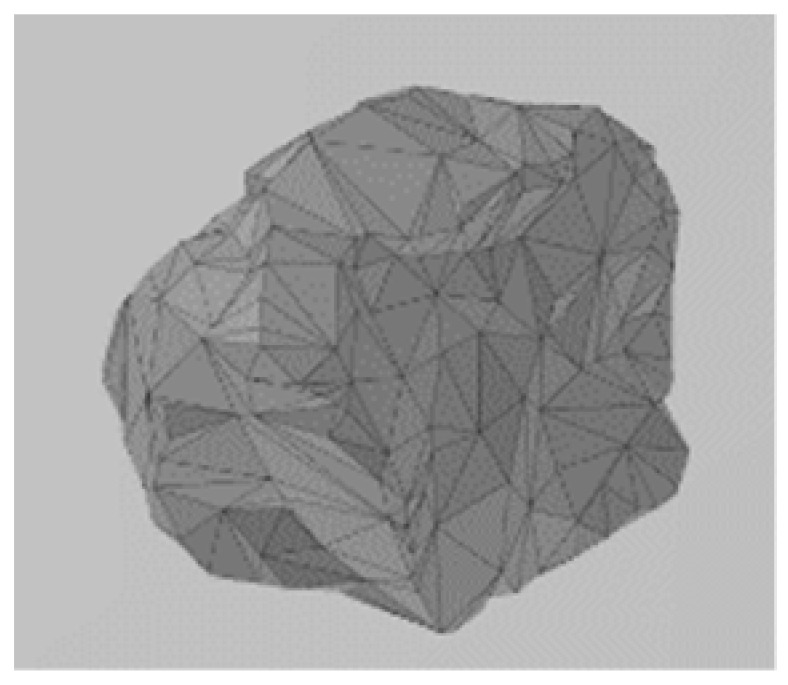
Clump in DEM	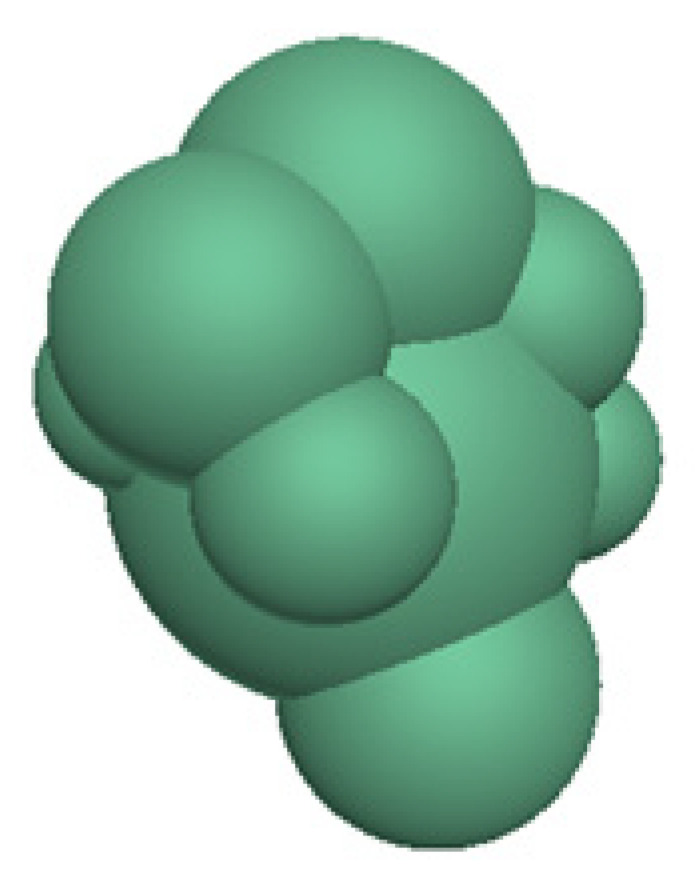	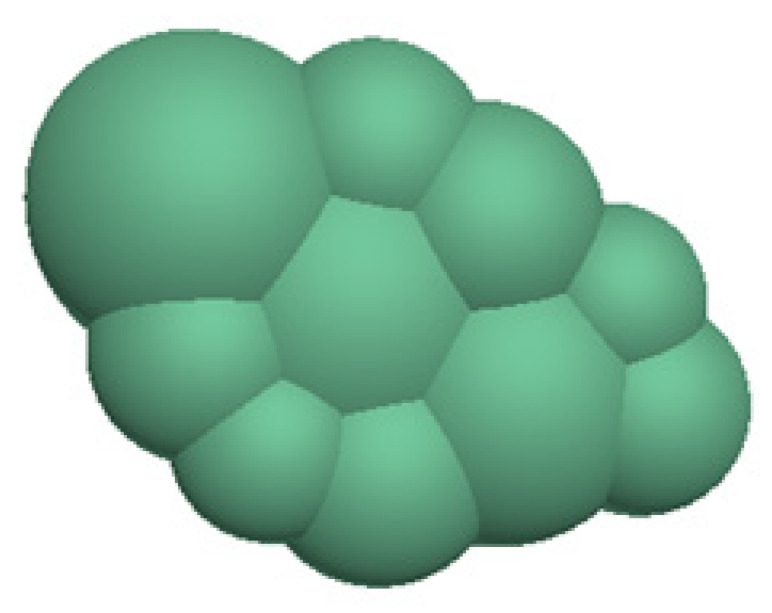	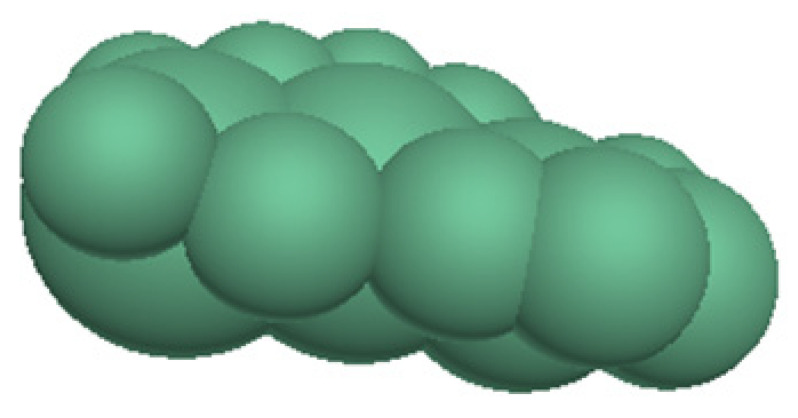	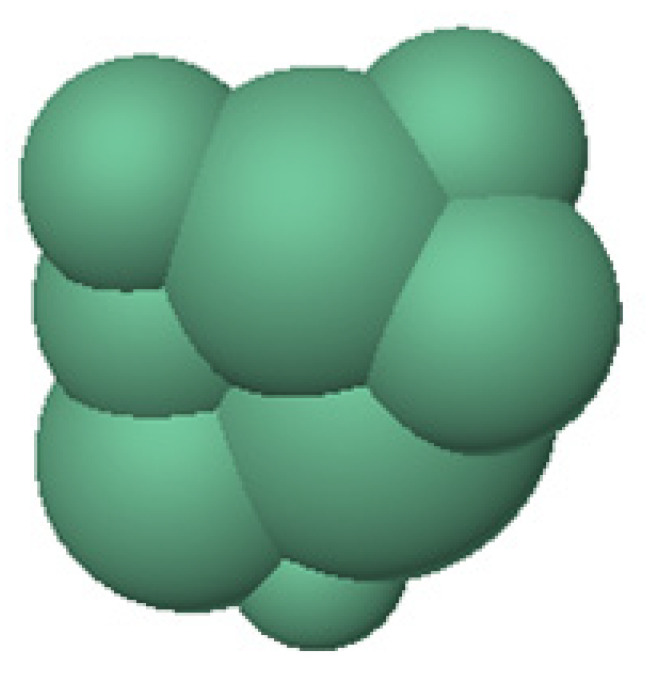	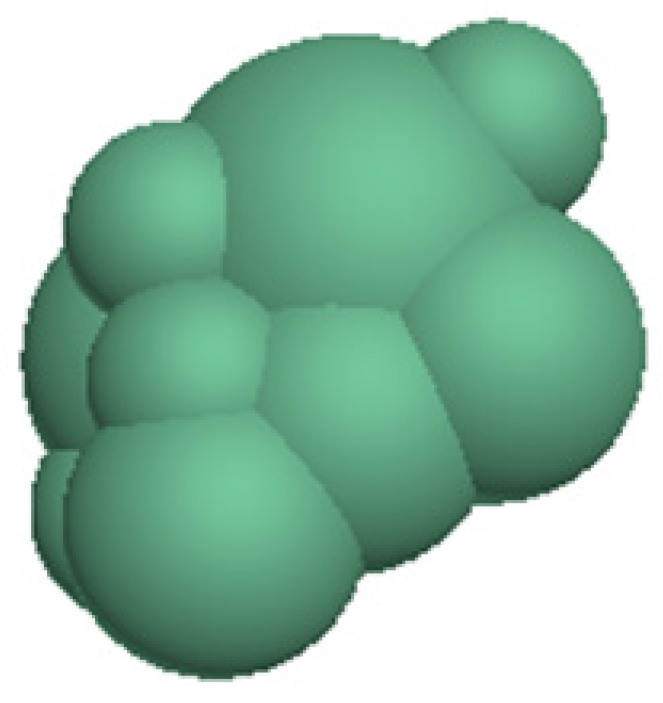

**Table 2:** Ballast model parameters.

Parameter	Value
Normal contact stiffness between ballast particle clumps/(N·m^−1^)	1 × 10^8^
Shear contact stiffness between ballast particles/(N·m^−1^)	1 × 10^8^
Normal contact stiffness between ballast and wall/(N·m^−1^)	1 × 10^9^
Shear contact stiffness between ballast and wall/(N·m^−1^)	1 × 10^9^
Friction coefficient of ballast particles	0.55
Coefficient of friction between ballast and wall	0.5
Density of ballast particles (kg·m^−3^)	2600

**Table 3:** Bearing stiffness of experiment and simulation.

	Experiment	Simulation	Difference
Bearing stiffness/(kN·mm^−1^)	184	173.63	6.1%

**Table 4:** Parameters of vehicle/track coupling model.

Parameter	Value
Car body mass (loaded)/kg	91,838
Mass of the bolster/kg	745
Mass of the side frame/kg	497
Wheelset mass/kg	1171
Stiffness coefficient of the primary suspension along the *X*-axis/(N·m^−1^)	13 × 10^6^
Stiffness coefficient of the primary suspension along the *Y*-axis/(N·m^−1^)	11 × 10^6^
Stiffness coefficient of the primary suspension along the *Z*-axis/(N·m^−1^)	35 × 10^6^
Stiffness coefficient of the secondary suspension along the *X*-axis/(N·m^−1^)	3.13 × 10^6^
Stiffness coefficient of the secondary suspension along the *Y*-axis/(N·m^−1^)	3.13 × 10^6^
Stiffness coefficient of the secondary suspension along the *Z*-axis/(N·m^−1^)	4.24 × 10^6^
Wheel radius/m	0.42
Rail mass per meter/kg	74.4
Sleeper mass/kg	360
Ballast mass/kg	560
Vertical pad stiffness/(N·m^−1^)	120 × 10^6^
Vertical ballast stiffness/(N·m^−1^)	100 × 10^6^
Vertical subgrade stiffness/(N·m^−1^)	100 × 10^6^
Sleeper spacing/m	0.6
Gauge/m	1.435

## Data Availability

Data are contained within the article.
